# Integrated treatment of hepatitis C virus infection among people who inject drugs: study protocol for a randomised controlled trial (INTRO-HCV)

**DOI:** 10.1186/s12879-019-4598-7

**Published:** 2019-11-08

**Authors:** Lars T. Fadnes, Christer Frode Aas, Jørn Henrik Vold, Christian Ohldieck, Rafael Alexander Leiva, Fatemeh Chalabianloo, Svetlana Skurtveit, Ole Jørgen Lygren, Olav Dalgård, Peter Vickerman, Håvard Midgard, Else-Marie Løberg, Kjell Arne Johansson, for the INTRO-HCV Study Group, Christer Frode Aas, Vibeke Bråthen Buljovcic, Fatemeh Chalabianloo, Jan Tore Daltveit, Silvia Eiken Alpers, Lars T. Fadnes, Trude Fondenes Eriksen, Per Gundersen, Velinda Hille, Kristin Holmelid Håberg, Kjell Arne Johansson, Rafael Alexander Leiva, Siv-Elin Leirvåg Carlsen, Martine Lepsøy Bonnier, Lennart Lorås, Else-Marie Løberg, Mette Hegland Nordbotn, Cathrine Nygård, Maria Olsvold, Christian Ohldieck, Lillian Sivertsen, Hugo Torjussen, Jørn-Henrik Vold, Jan-Magnus Økland, Tone Lise Eielsen, Nancy Laura Ortega Maldonado, Ewa Joanna Wilk, Ronny Bjørnestad, Ole Jørgen Lygren, Marianne Cook Pierron, Olav Dalgard, Håvard Midgard, Svetlana Skurtveit, Peter Vickerman

**Affiliations:** 1 Bergen Addiction Research Group, Department of Addiction Medicine, Haukeland University Hospital, Bergen, Norway; 2 Department of Global Public Health and Primary Care, University of Bergen, Bergen, Norway; 3 Department of Medicine, Haukeland University Hospital, Bergen, Norway; 4 Norwegian Centre for Addiction Research, University of Oslo, Oslo, Norway; 5 Department of Mental Disorders, Norwegian Institute of Public Health, Oslo, Norway; 6 ProLAR Nett, Oslo, Norway; 7 Department of Infectious Diseases, Akershus University Hospital, Lørenskog, Norway; 8 Institute for Clinical Medicine, University of Oslo, Oslo, Norway; 9 University of Bristol, Bristol, UK; 10 Department of Gastroenterology, Oslo University Hospital, Oslo, Norway; 11 Department of Clinical Psychology Medicine, University of Bergen, Bergen, Norway; 12 Division of Psychiatry, Haukeland University Hospital, Bergen, Norway

**Keywords:** Chronic hepatitis C, Opiate substitution treatment, Integrated health care, Substance abuse treatment centres

## Abstract

**Background:**

A large proportion of people who inject drugs (PWID) living with hepatitis C virus (HCV) infection have not been treated. It is unknown whether inclusion of HCV diagnostics and treatment into integrated substance use disorder treatment and care clinics will improve uptake and outcome of HCV treatment in PWID. The aim is to assess the efficacy of integrating HCV treatment to PWID and this paper will present the protocol for an ongoing trial.

**Methods:**

INTRO-HCV is a multicentre, randomised controlled clinical trial that will compare the efficacy of integrated treatment of HCV in PWID with the current standard treatment. Integrated treatment includes testing for HCV, assessing liver fibrosis with transient elastography, counselling, treatment delivery, follow-up and evaluation provided by integrated substance use disorder treatment and care clinics. Most of these clinics for PWID provide opioid agonist therapy while some clinics provide low-threshold care without opioid agonist therapy. Standard care involves referral to further diagnostics, treatment and treatment follow-up given in a hospital outpatient clinic with equivalent medications. The differences between the delivery platforms in the two trial arms involve use of a drop-in approach rather than specific appointment times, no need for additional travelling, less blood samples taken during treatment, and treatment given from already known clinicians. The trial will recruit approximately 200 HCV infected individuals in Bergen and Stavanger, Norway. The primary outcomes are time to treatment initiation and sustained virologic response, defined as undetectable HCV RNA 12 weeks after end of treatment. Secondary outcomes are cost-effectiveness, treatment adherence, changes in quality of life, fatigue and psychological well-being, changes in drug use, infection related risk behaviour, and risk of reinfection. The target group is PWID with HCV diagnosed receiving treatment and care within clinics for PWID.

**Discussion:**

This study will inform on the effects of an integrated treatment program for HCV in clinics for PWID compared to standard care aiming to increase access to treatment and improving treatment adherence. If the integrated treatment model is found to be safe and efficacious, it can be considered for further scale-up.

**Trial registration:**

ClinicalTrials.gov.no. NCT03155906.

## Background

Hepatitis C virus (HCV) is endemic among people who inject drugs (PWID) [1]. Globally 40% of the HCV disease burden is attributable to injecting drug use and in Western Europe this fraction is 64%. Recently, highly effective direct-acting antiviral (DAA) treatment of HCV infection became available, prompting the World Health Organisation (WHO) to develop a global strategy to eliminate HCV by 2030. However, as PWID represent the majority of HCV patients in high income countries there are challenges particular to this group that must be addressed if elimination is to be achieved [2].

Chronic HCV infection is a slowly progressive disease that may lead to liver cirrhosis and subsequent complications, including hepatocellular carcinoma (HCC), liver failure and early deaths [3, 4]. Among HCV-infected people with opioid dependence, one-third develops advanced liver disease within three decades [5] and within this group, liver disease and drug overdose becomes equally common causes of death among individuals over the age of 50 years [6]. Reports from opioid agonist therapy (OAT) clinics in Norway indicate that more than half of patients receiving OAT have chronic HCV infection [7]. This represents an ageing cohort often at high risk of developing advanced liver disease [8]. Ongoing injecting risk behaviour with risk of onwards HCV transmission is also prevalent in this population [9]. Thus, reaching these individuals with HCV treatment is of critical importance both to reduce HCV disease burden and HCV transmission.

Until 2014, HCV treatment was interferon-based with low efficacy, considerable adverse effects and accordingly low treatment uptake particularly among PWID [10]. The perceived high risk of reinfection following successful treatment may also have represented a barrier to HCV treatment in this population [9]. The current availability of tolerable and highly efficient all-oral DAA treatment has changed the HCV care paradigm and led to significant therapeutic optimism. However, The World Hepatitis Summit in 2017 expressed concern for the lack of integrated approaches, acknowledging the need for new models of patient-centred service delivery and recognised the need for integrated collaborative approaches [11]. To achieve this, new models of care must be developed. Models focusing on interdisciplinarity, availability and accessibility, with decentralised clinics and frequent follow-ups have been suggested to be effective [12–14]. However, reviews have called for further evidence on the effect of integrated treatment for people with substance use disorders [15, 16].

In Western Norway, more specifically in the cities of Stavanger and Bergen, OAT treatment is administered in an integrated treatment and care model with follow-up by physicians, nurses, social workers and psychologists on nearly a daily basis with observed intake of OAT medications [17]. PWID not receiving OAT are offered care at municipal low-threshold health and care clinics. Thus, the models already in place could be a well-suited platform to evaluate the effect of integrated HCV care to PWID.

### Objectives

This paper presents the protocol of the INTRO-HCV study. The primary objective of the INTRO-HCV study is to compare the efficacy of integrated treatment of HCV in PWID with the current standard treatment. Integrated treatment includes testing for HCV, assessing liver fibrosis with transient elastography, counselling, treatment delivery, follow-up and evaluation provided by integrated substance use disorder treatment and care clinics. Most of these clinics for PWID provide OAT while some clinics provide low-threshold care without OAT. Standard care involves referral to further diagnostics, treatment and treatment follow-up given in a hospital outpatient clinic with equivalent medications.

Secondary objectives are comparison of treatment adherence between the integrated and standard arms, assessment of changes in quality of life, fatigue and psychological well-being before and after HCV treatment, as well as changes in drug use, infection related risk behaviour, and risk of reinfection among those with sustained virologic response (SVR). Further, we will estimate cost-effectiveness of integrated HCV care and incidence and prevalence of HCV infection among PWID in Bergen and Stavanger to assess population effects of scaling up treatment.

## Methods

### Study design

This is a multicentre, randomised controlled clinical trial.

### Study settings and participants

The target group will be PWID receiving integrated substance use disorder treatment and care from involved clinics in Bergen and Stavanger who are chronically infected with HCV and eligible for treatment according to national guidelines.

Department of Addiction Medicine at Haukeland University Hospital in Bergen have adopted an integrated treatment and care model for PWID receiving OAT. In Bergen, OAT outpatient clinics have been established in each district where the patients are followed up by health and social workers on a nearly daily basis with observed intake of the OAT medications such as buprenorphine or methadone [17]. Every month, the OAT clinics have a total of 6000 visits among the approximately 500 patients. This group of patients have a large morbidity burden and have to a limited extent been able to get access to other standard health care. Each of the OAT outpatient clinics is staffed by a consultant and a physician/ specialist registrar in addiction medicine in addition to nurses, social workers, and psychologists. A close collaboration has been established between the Department of Addiction Medicine and the Agency for addiction and mental health in Bergen municipality, who is responsible for the care in several of the primary health clinics for PWID in Bergen. Clinics for PWID in the Stavanger area have a relatively similar structure and are responsible for the OAT treatment of approximately 460 patients within their area. People receiving OAT in Norway are generally prescribed buprenorphine or methadone, to some degree additional benzodiazepines but rarely other opioids. The treatment model in Bergen and Stavanger is a well-suited platform to test integration of HCV treatment aiming to improve the health and life span of a vulnerable group, and at the same time gathering knowledge which traditionally have been difficult to obtain.

### Eligibility criteria

For the randomised trial, inclusion will be based on the following criteria

- PWID receiving OAT or low-threshold care from an included clinic with follow-up on weekly basis
- Chronically infected with HCV (HCV RNA positive measured at least twice over 6 months)
- Eligible for treatment according to national guidelines (criteria specified below)
- Giving informed consent

Enrolment started May 2017. At that time, eligibility for treatment according to Norwegian guidelines was defined as follows:

- Genotype 1 and 4 infection independent of stage of liver fibrosis.
- Genotype 2 and 3 infection with significant liver fibrosis.

Transient elastography (FibroScan) will be used in the screening of significant fibrosis defined as measurements above 7.0 kPa [18, 19].

Thus, some patients needed to wait some months after HCV diagnosis before initiating treatment. From 1st of February 2018, all patients with chronic HCV were eligible for treatment according to the updated national (independent of stage of liver fibrosis).

The following exclusion criteria will be used:

- HIV co-infection
- Severe extrahepatic HCV manifestations (e.g. cryoglobulinemia)
- Membranoprolifereative glomerulonephritis (MPGN) or renal failure (estimated glomerular filtration rate < 30 ml/min/1.73 m^2^)
- Decompensated liver disease (Child-Pugh score > 6 points, class B and C)

Encephalopathy in Child-Pugh will be assessed according to the West Haven criteria [20].

- Currently receiving treatment for HCV

### Interventions

Participants randomised to standard care are first assessed and diagnosed at the integrated care clinics for PWID by nurse and physician and when found in need of treatment, are referred to a hospital clinic for further assessment and treatment. The standard care generally involves being referred to the medical outpatient clinic. The clinic will send a letter informing on time of appointment usually some weeks later and could involve some additional blood samples and imaging before initiating DAA treatment. Subsequently, an electronic prescription is given in a consultation where treatment is initiated. The medications given are in principle equivalent in the two trial arms. Most will receive medication combinations of either elbasvir/grazoprevir 50/100 mg or sofosbuvir/ledipasvir 400/90 mg (for genotype 1 and 4) or sofosbuvir/velpatasvir 400/100 mg (for genotype 2 and 3) – sometimes also in combination with voxilaprevir 100 mg or ribavirin 200 mg due to specific clinical indications. Glecaprevir/pibrentasvir 100/40 mg may also be used in special cases. Patients are generally given follow-up consultations every 4 weeks during treatment and a post-treatment assessment 12 weeks after completed treatment. Typically, this involves a total of 4–5 consultation visits at the hospital.

Participants randomised to integrated treatment are given integrated assessment and treatment at the clinics for PWID (by nurse and physician). For those found to be in need of treatment, medications are made available by a nurse that gives follow-up for OAT or low-threshold care for PWID. Follow-up during treatment is given in parallel with delivery of OAT treatment and other care. At 12 weeks post treatment, a research nurse takes blood samples to assess the treatment effect.

The differences between the delivery platforms in the two trial arms involve the following main aspects:

- Need of travel: Standard care involves transportation to a hospital clinic – a distance that ranges from 1 to about 25 km in distance (with potential travel costs) while integrated treatment does generally not require need for additional travel
- Need to come to specific appointment times: Standard treatment is generally given based on appointments while integrated treatment is generally given in a drop-in approach
- Frequency of blood samples during treatment is mainly depending on clinical indication (e.g. suspected adverse effects or complications). Less blood sampling may reduce discomfort
- Time used and potential for overlapping appointments: For standard treatment there is a higher risk that the appointment time overlap with other important activity including other health care, while for integrated treatment appointment time is generally planned together with patient
- Need to meet new clinicians for health care: In integrated care clinics, follow-up is generally done by already familiar contact nurse and physician providing regular follow-up. Standard treatment is given in hospital clinic by clinicians often unfamiliar to the patients

### Outcomes

Primary outcome measures are

- Sustained virologic response will be assessed by undetectable HCV RNA at 12 (range 10–14) weeks after completed treatment. The virologic blood samples in Bergen will be analysed at Department for microbiology (accredited by ISO-standard 15,189) after being centrifuged at each study clinic before transfer
- Time to treatment initiation after diagnosing HCV in need of treatment (in line with national guidelines). This will be assessed with time-to-event analyses. We also plan to present stratified analyses on patient-related delay and system-related delay

Secondary outcome measures are

- Treatment adherence assessed by proportion of doses observed being taken in intervention group and reported obtainment from pharmacies of the prescribed drugs combined with self-reported questions on adherence collected at 4, 8 (and 12 for treatment recommended beyond 8 weeks) weeks after treatment initiation. The self-reported questions categorise adherence during last four weeks into rarely, sometimes but less than half of the doses, between 50 and 80% of the doses, more than 80% of the doses, and always (95% of doses or more). These outcome measures will be collected at the OAT/PWID clinics
- Reinfection will be defined as HCV RNA recurrence following SVR. Incidence rates will be calculated using person-time techniques assuming a Poisson distribution. All patients achieving SVR will be assessed for reinfection 3 months after treatment and then annually. HCV RNA positive samples will be further analysed with a quantitative HCV RNA count, HCV genotyping. Relapse is defined as presence of the same virus strain at the time of diagnosis and at the end of treatment. Anti-HIV and use of other drugs will also be assessed
- Changes in quality of life will be assessed with the questionnaire EQ-5D-5 L (https://euroqol.org/eq-5d-instruments/) in addition to a self-reported question on happiness on a 0 to 10 scale at 12 weeks after treatment compared to before treatment
- Changes in fatigue will be assessed with the Fatigue Symptom Scale at 12 weeks after treatment compared to before treatment
- Changes in psychological well-being will be assessed with the Norwegian validated translation version of Hopkins Symptom Checklist (SCL-10) at 12 weeks after treatment compared to before treatment
- Assessment of changes in substance use will be assessed with self-reported use of the following drug categories the last 30 days, the last 12 months and ever: Alcohol, tobacco, cannabis, amphetamines, cocaine, heroin, other opioids not prescribed by physician, benzodiazepines or z-hypnotics, hallucinogens, solvents and gamma hydroxybutyrate (GHB), anabolic steroids and other drugs at 12 weeks after treatment compared to before treatment
- Assessment of changes in injecting risk behaviour will be performed with questions assessing sharing of needles and other user equipment before and after HCV treatment at 12 weeks after treatment compared to before treatment
- Health provider costs by using an ingredients approach, where quantities used and the value (or price) of each unit is estimated in the both trial arms. Health care costs will be used in a health economic evaluation, where both costs per cured patient and per Quality Adjusted Life Year (QALY) gained will be estimated.

### Sample size

We expect higher rates of treatment success in the intervention arm receiving integrated treatment compared to the standard arm. The power calculation is based on the following assumptions:

- The power is set at 90% with a two-sided alpha (α) error of 5%.
- Comparison of SVR at 12 weeks.
- Up to 33% lost to follow-up at 12 weeks after treatment.
- Equal proportions between the groups, 30% higher rates of successfully cured in integrated compared to standard treatment (e.g. 50% in the standard arm and 80% in the intervention arm).
- Statistical power calculations in Stata.

Based on these assumptions, 87 persons are required in intervention arm and 87 persons in the control arm. Reducing the differences in rates of successfully cured to 25% while reducing power to 80%, 99 persons in each arm would be required (or 66 persons if assuming no lost-to-follow-up).

### Recruitment

All PWID receiving OAT treatment or low-threshold care from included clinics will be considered the reference target population. As part of an annual clinical assessment, patients will be informed about the study and asked for consent to participation. All patients in target population will be offered annual clinical assessment and study participation. For those giving informed consent, an extended clinical assessment will be offered and those diagnosed with chronic HCV will be randomised for the intervention.

### Allocation and blinding

We will use block randomisation with a 1:1 ratio using blocks of 10 to ensure relatively similar distribution between both arms throughout different time periods of the trial. Randomisation will be electronically registered. The randomisation will be stratified by site/county (site 1: Bergen/Hordaland and site 2: Stavanger/ Rogaland).

Even though complete blinding is regarded as difficult and would probably come at a cost of reduction in external validity [21], there will be some degree of blinding/masking. Randomisation will be disclosed to physician and other health care staff providing treatment and follow-up for PWID, but not to research nurses conducting data collection for outcomes. Patients will be informed of which intervention arm they are randomised to and the form of follow-up they will receive, but not on other follow-up alternatives they do not receive and what the hypotheses for the study are.

### Data collection and management

**Fig. 1:**
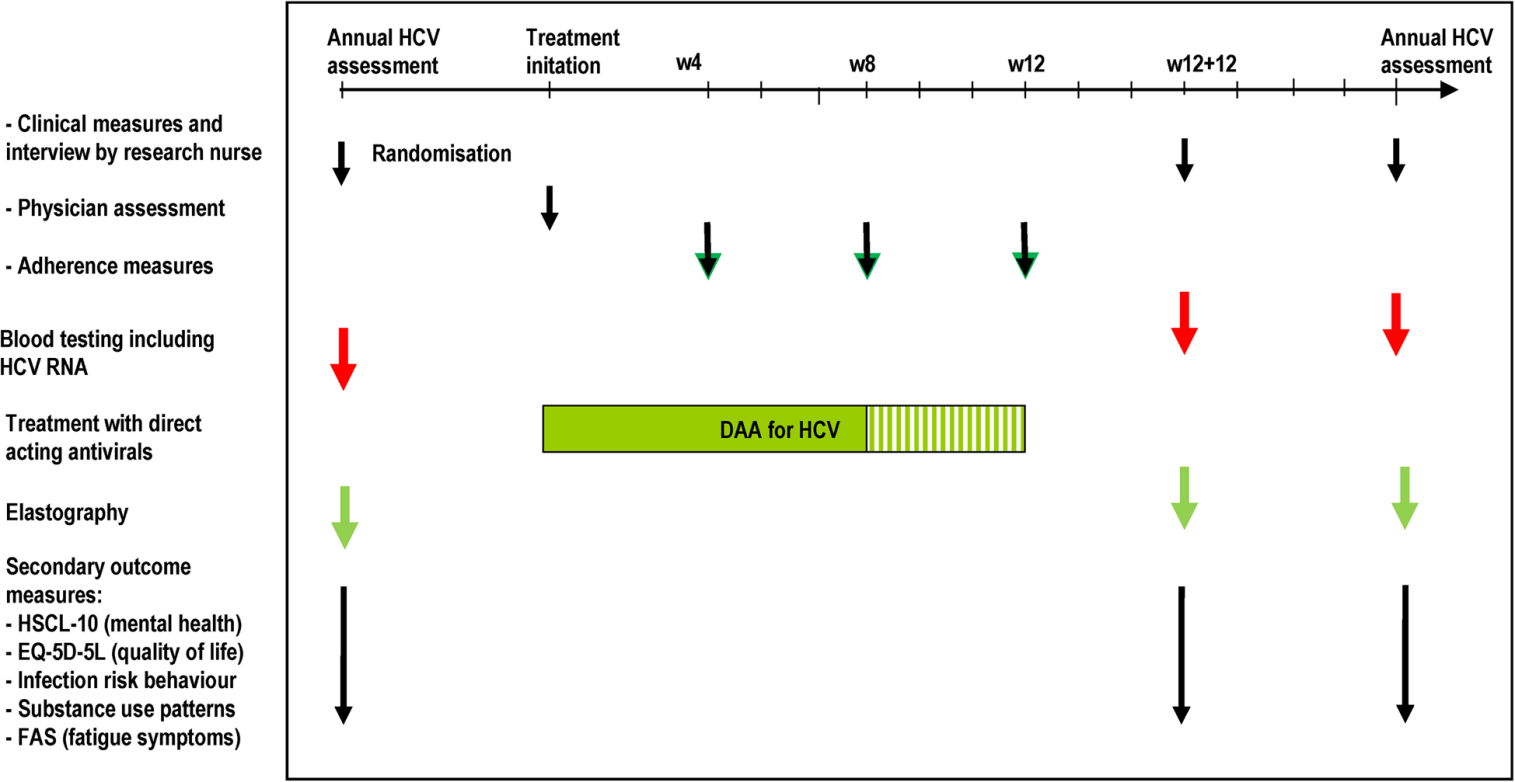
Overview of follow-up for the study * The arrows are indications of when the various measured are timed

Data collection and follow-up will be given in line with Table 1 and Fig. 1.

**Table 1:** Flow chart of the study outlining follow-up visits and assessments at each visit

	Screening 1 (part 1)	Screening 2 (part 2)	Treatment initiation or referral to standard treatment (physician)	Treatment follow-up (4 weeks after initiation)	Treatment follow-up (8 weeks after initiation)	Treatment follow-up (12 weeks after initiation)	Effect assessment (12 weeks after completion)	Annual follow-up
Acceptable time shift (days)				± 7	± 7	± 7	± 14	± 60
Physician assessment			X				X	X
Research nurse assessment	X	X					X	X
- Informed consent	X							
- Eligibility assessment	X	X	X					
- Follow-up by staff delivering treatment and providing information (several times weekly)			X	X	X	X		
- Clinical assessment	X	X	^b^	^b^	^b^	^b^		X
- Counselling on prevention			X	X	X	X	X	X
Elastography^a^		X					(X)	X
Lab tests								
- Viral testing	X						X	X
Full blood count and transaminases	X			^b^	^b^	^b^	X	X
HSCL-10 (mental health)	X						X	X
FAS (fatigue symptoms)	X						X	X
EQ-5D-5 L (quality of life)	X						X	X
Infection risk behaviour	X						X	X
Substance use patterns	X						X	X
HCV genotyping and viral load	X						X	X

^**a**^Elastography after treatment will be assessed in Bergen and not in Stavanger due to elastography availability only in hospital clinic and not outpatient clinic

^b^Additional tests will be taken on clinical indication

The blood samples for the primary outcome measures will be collected at the OAT/PWID clinics for participants randomised to integrated care and at the hospital outpatient clinic for the participants randomised to standard care. However, for patients who do not come for assessment at the hospital clinic 12 weeks after treatment, efforts will be made to collect these blood samples at the OAT/PWID clinic. For the remaining outcome measures, these will be measured at the OAT/PWID clinics by research nurses through a structured interview for both participants randomised to standard and integrated treatment.

### Analyses and statistical methods

A detailed plan for analysis will be developed before data export and analysis. We will in this section outline some of the principles which will be used to guide decisions during data analysis. Analysis methods will follow the CONSORT and SPIRIT guidelines as far as possible [22–24]. All tests will be two-sided. Descriptive results and efficacy estimates will be presented with 95% confidence intervals. The statistical significance is set at *p* < 0.05. Potential confounders may be considered for adjustment if they are imbalanced at baseline (with assumed meaningful differences). Missing data will be considered, and appropriate imputations based on pre-defined assumptions will be done when necessary (as described in detailed plan of analysis). Categorical variables will be summarized as percentages and continuous variables as medians with interquartile ranges or means with standard deviation for variables with a Gaussian distribution. The main outcomes will be analysed with time-to-event analysis (Cox regression and Kaplan-Meier plots) and generalised linear models/logistic regression. A separate health economic evaluation will assess the incremental cost-effectiveness ratio of the intervention arm compared to the control arm. A Markov model will be used, and the analysis will be exhibited in TreeAge. Results will be presented as cost per QALYs of integrated versus standard HCV care.

### Potential harms and data monitoring

Those who participate in the study will be randomised to one of two different follow-up programs. It is possible that the treatment follow-up in the integrated treatment is inferior in quality to the standard treatment. Several measures have been taken to ensure sufficient quality of the treatment follow-up and to safeguard through detection of potential severe adverse effects (SAEs). It is also possible that the integrated treatment will lead to better treatment adherence and response and thus less morbidity.

There is a risk of hepatic decompensation during treatment of patients with cirrhosis and portal hypertension [25]. To reduce this risk, patients with decompensated cirrhosis (Child Pugh class C or D) will be excluded. Further, there is a small risk of reactivation of hepatitis B virus among patients with occult infection after treatment for HCV and potentially liver failure unless properly assessed [26]. However, patients with a possible occult hepatitis B infection (anti-HBc positive, anti-HBs negative and HBsAg negative) will be closely followed up by weekly blood sampling during the first 4 weeks of treatment including liver enzyme assessments and HBV DNA (PCR) in order to being able to identifying reactivation in early stages.

All grade 3 and 4 adverse effects are considered as SAE and will be reported as such. For the evaluation of safety, all SAEs occurring during the trial follow-up period will be reported on the Clinical Report Form using a national registration form for medication related side effects. A copy of the form will be sent to RELIS Vest in addition to a copy sent to the clinical coordinators and steering committee reviewing all cases. Adverse effects grade 1 or 2 will be assessed through a questionnaire.

All SAEs will be followed until resolution or until a stable clinical end-point is reached. All measures required for SAE management and the ultimate outcome of the SAE will be recorded.

Similarly, there is potentially a risk for development of HCV resistant to direct acting antiviral medications, particularly among patients not completing treatment. Efforts will be made to avoid treatment failure and aiming for a secondary curative treatment among those failing first treatment. There will not be an independent data monitoring committee. The study coordination unit will be responsible to ensure adherence to the protocol, quality of the study and ethical conduct.

## Discussion

The research project will improve knowledge on the impact on health care organisation and structure on patient outcomes. We will assess a platform aiming for easier access to treatment of HCV among PWID who have been difficult to reach with ordinary health services. Integrated treatment models may be an opportunity to increase adherence to HCV treatment and evaluate collaborative care in light of access to direct acting antiviral medications. The treatment platform could reduce the burden of HCV among this patient population and could contribute to HCV elimination. We will test this with a randomised controlled trial done in parallel with an observational study assessing HCV reinfections, other infections including HIV, and liver disease severity.

Our trial involves some limitations and several strengths. For the trial it is difficult to ensure complete blinding, however, we will aim for blinding of data collectors and some degree of blinding for participants. The study is also funded from public sources ensuring independency. We also have a biological primary outcome. Thus, substantial information biases are considered unlikely. The study is individually randomised minimizing potential confounding. The study population of PWID will include a large proportion struggling to obtain standard care. This will not be applicable to all groups of people with substance use disorders. For people with substance use disorders with higher levels of functioning and less need for regular treatment follow-up, the need for treatment integration might not be as relevant. The study setting is also very suitable to test out treatment integration. The time prior to change in the national guidelines for treatment of HCV made it necessary for some people to delay HCV treatment initiation. This might impact negatively on the treatment outcomes, but probably is balanced between the arms. Further, most of the participants will be treated after the guideline changes where treatment is now available independent of stage of liver fibrosis. Subsequently, we assume that this effect will not be substantial. The study might be well suited to assess some biological effects of HCV infection such as to which extent HCV contributes to fatigue, reduced quality of life, occurrence of mental disorders and continued drug use. The study size should be sufficient to answer the primary objectives with high precision and is assumed to have adequate precision also for secondary objectives. In terms of safety, the frequent follow-up of PWID could improve detection of potential adverse effects of the applied medications. One can see trials on a continuity from efficacy trials optimising internal validity through homogeneous population to effectiveness trials optimising external validity with a more heterogeneous population. This trial is tending towards the effectiveness side of the spectrum. An alternative design could have been a stepped-wedge trial which could have required slightly smaller sample sizes and would also be well opted to measure effectiveness of integrated treatment. However, we expect to have enough participants to meet the sample size requirements. On the other side, stepped-wedged trials generally take longer time to conduct due to the time delay. In addition, our more conventional trial design is less vulnerable to confounding from time trends.

Our assessment will also inform future health policy on efficiency by using standard methods of cost-effectiveness analysis taking both the expected effectiveness and provider costs of the intervention into consideration. Even though there is evidence on the cost-effectiveness of standard DAA in HCV treatment regimens; there is limited evidence for the cost-effectiveness of alternative scale-up scenarios taking the complex behavior of PWID into account [27].

If the integrated treatment model for HCV in clinics for PWID compared to standard care is found to be safe and efficacious in terms of increasing access and improving treatment outcomes, this model could be considered for further scale-up.

## Data Availability

Trial outcome data is not yet available.

## Abbreviations

DAA: Direct-acting antiviral treatment
HCV: Hepatitis C virus
OAT: Opioid agonist therapy
PWID: People who inject drugs
QALY: Quality Adjusted Life Year
SAE: Severe adverse effects
SVR: ustained virologic response
